# In-vitro and in-vivo evaluation of a novel bioprosthetic pulmonary valve for use in congenital heart surgery

**DOI:** 10.1186/s13019-019-0830-1

**Published:** 2019-01-09

**Authors:** Jonas Rasmussen, Søren Nielsen Skov, Ditte Bruus Nielsen, Ida Lindhardt Jensen, Marcell Juan Tjørnild, Peter Johansen, Vibeke E. Hjortdal

**Affiliations:** 1 Department of Cardiothoracic and Vascular Surgery, Aarhus University Hospital, Palle Juul-Jensen Boulevard 99, 8200 Aarhus, Denmark; 2 Department of Clinical Medicine, Aarhus University Hospital, Aarhus, Denmark; 3 Department of Engineering, Aarhus University, Aarhus, Denmark

**Keywords:** Congenital heart surgery, Pulmonary valve, Artificial heart valve, Bioengineering

## Abstract

**Background:**

Management of congenital malformations of the pulmonary artery and valve can be challenging. The severity often demands early intervention, which is rarely definitive due to the natural growth and multiple surgeries may be required. An artificial valve made entirely from biodegradable materials that will serve as a bioscaffold for host recellularization would be an attractive solution for these patients. Such valves have been experimentally evaluated with various results. In this study, a simple valve design supported by an absorbable proximal stabilization ring is evaluated both in-vitro and in-vivo.

**Methods:**

From a 6.7 × 5.0 cm sheet of CorMatrix® tissue we created the valve as an inverted tubegraft with three sutured commissures. A non-closed ring of LactoSorb® basally supported the valve. The commissure height was 2 cm. Inserted as an interposition graft the valve was tested in an in-vitro model and an acute porcine model. Right ventricular and pulmonary artery pressures were recorded.

**Results:**

The in-vitro testing indicated a proper opening and closure function of valve at physiological simulated hemodynamic conditions. The in-vivo evaluation showed a peak right ventricular pressure of 38 mmHg and a peak pulmonary artery pressure of 27 mmHg and thereby a peak valve gradient of 11 mmHg. The pulmonary pressure wave demonstrated a dicrotic notch indicating competence of the valve.

**Conclusion:**

This new pulmonary valve made entirely from biodegradable tissue worked in an acute setting and displayed a good hemodynamic profile. The valve gradient observed is equal to or superior of today’s surgical treatment options.

## Background

Long lasting treatment of congenital malformations of the pulmonary artery and valve are challenging due to the growth of the heart. In the case of Tetralogy of Fallot, several strategies are used [1], and a majority of the patients will have to undergo multiple surgeries or catheter based interventions. The timing of the second surgery and potential replacement of the pulmonary valve is still explored and debated. Waiting for full growth may jeopardize the patient as the risk of a failing right ventricle, arrhythmias and sudden death increase with time [2, 3].

Therefore, an ideal surgical solution to pulmonary malformations would be early implantation of a valve that fits the newborn, but in the long term allows and accommodates growth of the valve along with the growth of the recipient.

Small intestine submucosal extra cellular matrix (SIS-ECM) is a decellularized bioscaffold made from porcine small intestine that is commercially available. This material should, amongst others, have the ability to be recellularized by the recipient and subsequent degeneration of the bioscaffold and thereby allow growth [4].

The SIS-ECM tissue has been used as a bio scaffold in numerous anatomic sites including cardiac structures and vessels both in experimental studies [5–7] as well as clinical use [8]. The results have been mixed, with positive findings but also various results have been reported regarding the degeneration of the SIS-ECM material [9, 10].

At our institution, the SIS-ECM tissue was evaluated in a tricuspid position as a tubegraft implanted in a chronic porcine model. Biomechanical and histological examinations were performed 6 month after implantation. The explanted SIS-ECM tissue was found to be stronger than the native valve tissue while histological results showed the initial acellular SIS-ECM material to be remodel with connective tissue and endothelialization [11].

In a study by Miller and colleagues [12] an artificial pulmonary valve was made by SIS-ECM and implanted into a total of six piglets. Initially, the valves were found to be well functioning with only trivial regurgitation and a peak valve gradient of 10.1 ± 2.5 mmHg. However, at 6 months follow up all animals had developed at least moderate regurgitation and an increased peak gradient across the valve of 36.3 ± 18.8 mmHg. The valves had increased in size, yet considerably less than the corresponding growth of the pig.

More innovation and evaluation are required before a reliable technique for making and using a SIS-ECM pulmonary valve can be considered as a generally reliable surgical treatment.

The previous issues with development of both valve stenosis and insufficiency need to be addressed. We know from aortic, mitral and tricuspid valve surgery that stabilization is beneficial.

In this study, we added a basal non-closed ring of LactoSorb® to the SIS-ECM valve. The LactoSorb® material does not promote excess foreign body reactions and in orthopedics it reabsorbs within 12 to 18 months after implantation [13].

We designed a non-closed ring to allow for optimal annular diameter sizing at the time of surgery. As the ring is not closed, with the two ends merely touching each other, it should not prevent dilatation and growth of the annular area as the recipient grows.

In this study we did a thorough in-vitro and acute in-vivo evaluation of an artificial pulmonary valve conduit made from SIS-ECM. The aim was to design a pulmonary valve with proximal valve stabilization and opportunity for intra-operative adjustment for the individual patient.

## Methods

### Study design

This study was carried out in three steps. First, we evaluated the in-vivo hemodynamics and the dimensions of a native pulmonary valve in a 60 kg pig. The size of the native pulmonary valve was used to properly size the new SIS-ECM valve. The valve was then evaluated in both a mechanical in-vitro model and then in a porcine in-vivo model.

### The valve

**Fig. 1:**
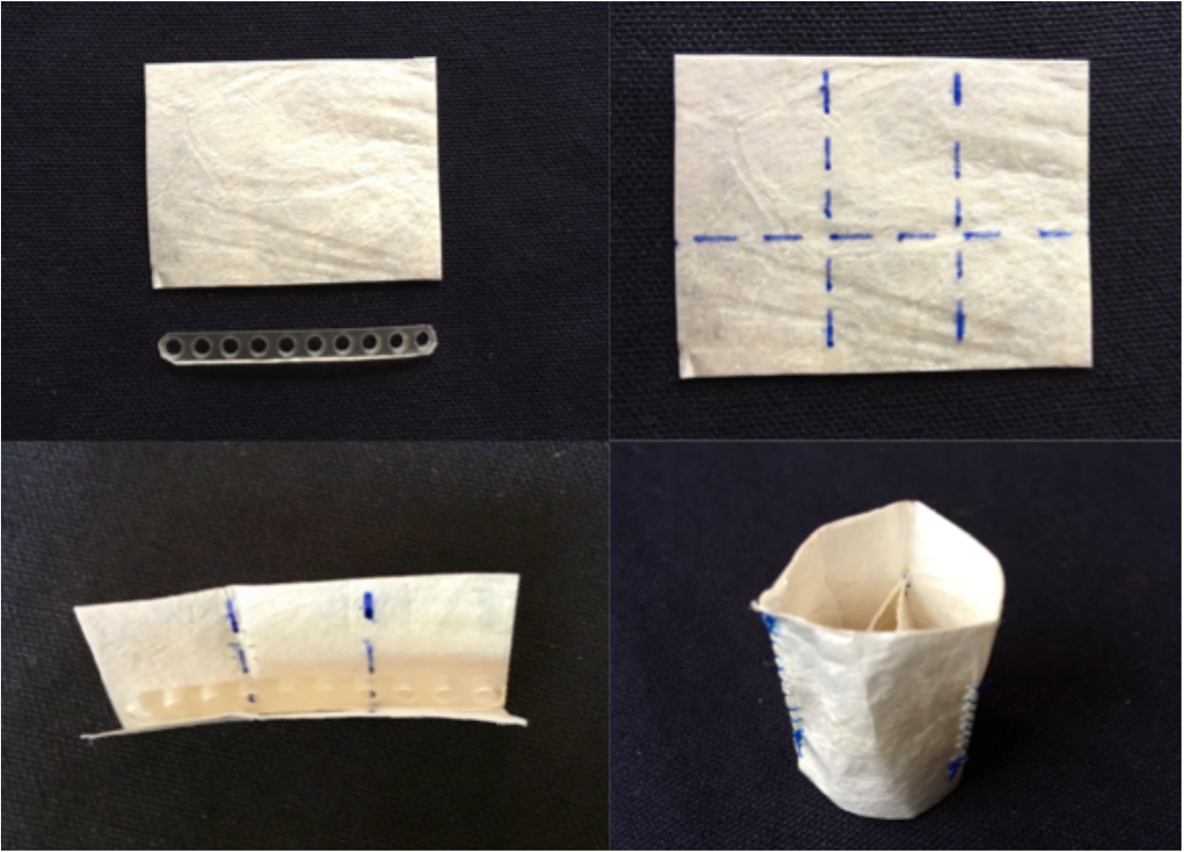
Step by step manufacturing of the valve. The dotted horizontal blue line seen in the upper right picture is the folding line. The dotted vertical lines are two of the suture lines creating the commissures. The last suture line, and thereby the third commissure, is made by suturing the two free edges tucked into each other. Note, the CorMatrix® becomes considerably more pliable once soaked

The valve design is illustrated in Fig. 1. The Valve was created using a rectangular piece of four-ply SIS-ECM (CorMatrix® Cardiovascular Inc., Alpharetta, GA, USA) measuring 6.7 × 5.0 cm. The sheet of SIS-ECM was folded horizontally to create one layer measuring 2 cm and one 3 cm in height. In the groove was placed a 6.3 cm long and 0.7 cm high strip of LactoSorb (Zimmer Biomet, Warsaw, IN, USA).

A tube was created by vertically rolling the folded sheet with a 4 mm overlap and then stitching the overlapping edges together with a 5–0 Prolene suture in a running fashion. Two additional vertical sutures across the two layers of SIS-ECM where then done in a fashion that would create three equally sized parts of the tube. This left a 1 cm free margin of the outer layer at what would become the distal part of the conduit valve. The valve had a circumference of 6.3 cm, a diameter of 2.0 cm, an inner height (corresponding to the commissure height) of 2.0 cm and outer height of 3.0 cm.

### Animals and operative procedure

Two mixed Yorkshire and Danish landrace female pigs with a body weight of 60 kg were used, one for the baseline measurements and one for the in-vivo testing. Both were bread and housed according to guidelines from the Danish Inspectorate of Animal Experimentation, whom also approved the experimental protocol of the present study. All surgical procedures were carried out at the Institute of Clinical Medicine, Aarhus University Hospital, Aarhus, Denmark, where proper facilities for animal experiments are available.

### Baseline measurements

After preparation of the animal the heart was exposed. Two microtip catheters (SPR-350S, Millar Instruments, Houston, TX, USA) were inserted, one in the right ventricle and one in the pulmonary artery. Simultaneous pressure data from both lumens were continuously recorded.

Finally, the pulmonary valve was excised with the right ventricle outflow tract and pulmonary artery attached for inspection and size measuring. These measures were used for sizing the new SIS-ECM valve.

### In-vitro model and testing

**Fig. 2:**
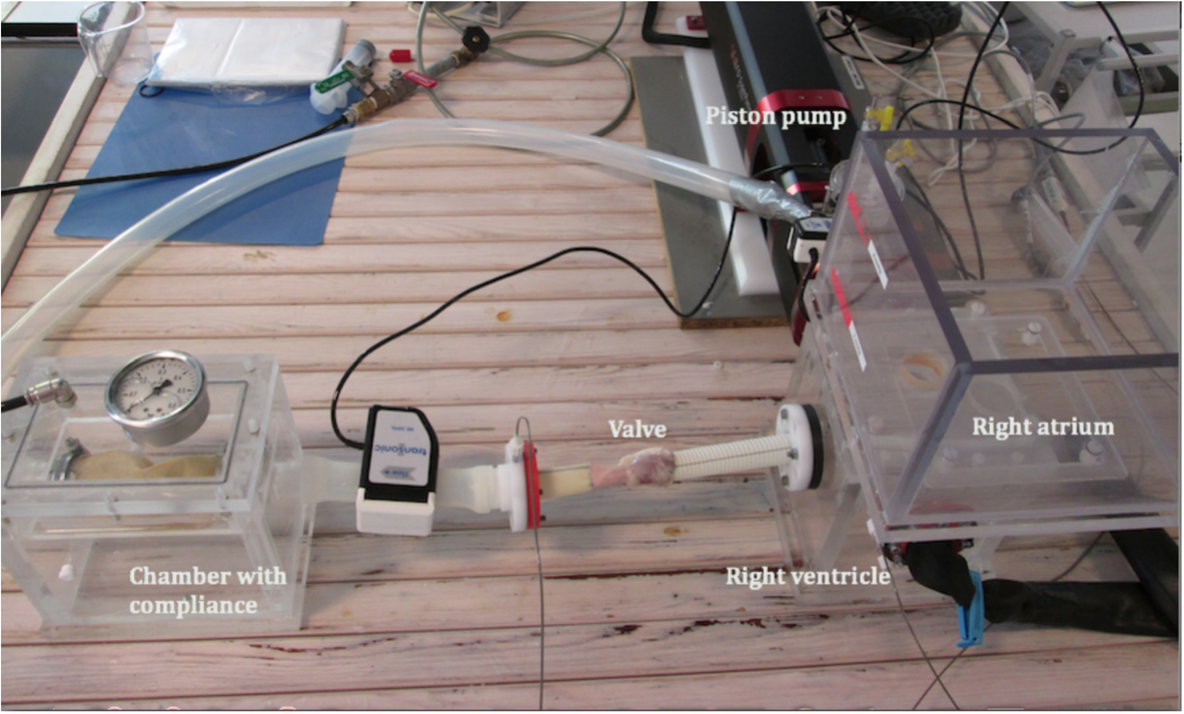
The in-vitro setup (not the SIS-ECM valve). In the upper left part of the picture the piston is seen. The piston is connected to the right ventricular chamber seen below the right atrium chamber. Attached to two pieces of Dacron tubes the valve is positioned. On the left, the compliance chamber is seen and from here a tube leads the fluid back to the right atrium chamber

All in-vitro testing was carried out in a pulsatile flow model using water as the test fluid (Fig. 2). Pulsatile flow and pressure were generated with an electromechanical piston pump (VSI Superpump, Vivitro Labs, Victoria, Canada).

The in-vitro testing was performed after interposition of the SIS-ECM valve between the ventricular and compliance chambers.

Pressures in the ventricular and compliance champers were recorded using the same microtip catheters as in all the in-vivo procedures.

### In-vivo testing

The pig was put under anesthesia and through a median sternotomy the heart was exposed and cardiopulmonary bypass was established for implantation.

The pre-manufactured SIS-ECM valve was soaked in saline water making it pliable. It was implanted as an end-to-end interposition graft. The three cusps were carefully inspected and found to move freely without any improper suture attachment.

Pressure measurements were collected using the same catheters and measuring equipment. The pulmonary artery catheter was placed distally for the SIS-ECM valve, but before the branching of the pulmonary artery. Echocardiography was performed to verify proper cusp movement and competence of the valve.

All results are reported as average values of 10 consecutive heartbeats followed by the standard deviation of the 10 beats.

## Results

### Baseline measurements

The native pulmonary valve of the first 60 kg pig was found to have a diameter of 2 cm. The peak right ventricular and pulmonary artery pressure were found to be 30.9 ± 0.1 mmHg and 30.4 ± 0.1 mmHg respectively, indicating virtually no valve gradient. Echocardiography revealed no valve abnormalities or insufficiency.

### In-vitro measurements

Tested in the in-vitro model, the valve performed well with proper closing and opening. At a peak right ventricular chamber pressure of 38.2 ± 0.3 mmHg a pulmonary artery pressure of 38.5 ± 0.3 mmHg were found. For practical purposes these results indicate no valve gradient. As seen in Fig. 4, a diacrotic notch on the pulmonary pressure curve was seen at the time of end-systole.

### In-vivo measurements

**Fig. 3:**
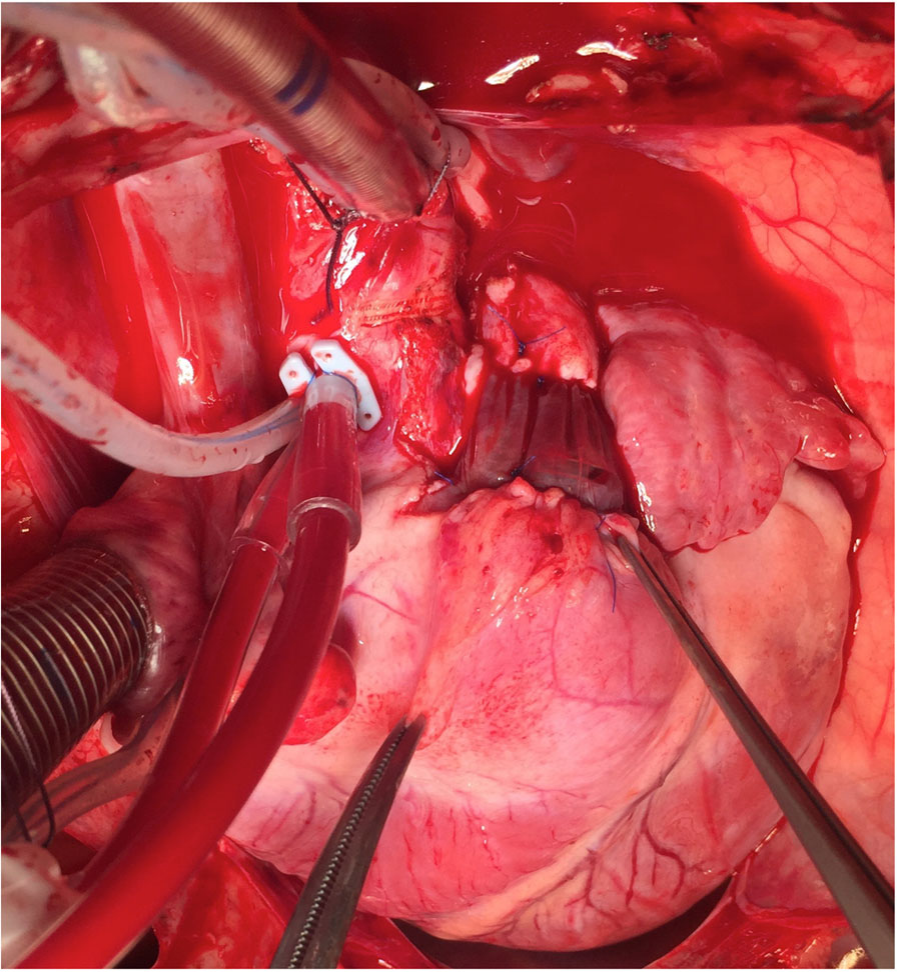
The valve implanted as an interposition graft in the pulmonary position. The right forceps are placed at the right ventricular outflow tract, just below the SIS-ECM valve. The left forceps grab the right ventricle

The valve was implanted and evaluated in the experimental in-vivo model (Fig. 3). Echocardiography demonstrated that all three cusps moved freely and uniformly. Only minor trace insufficiency was observed.

**Fig. 4:**
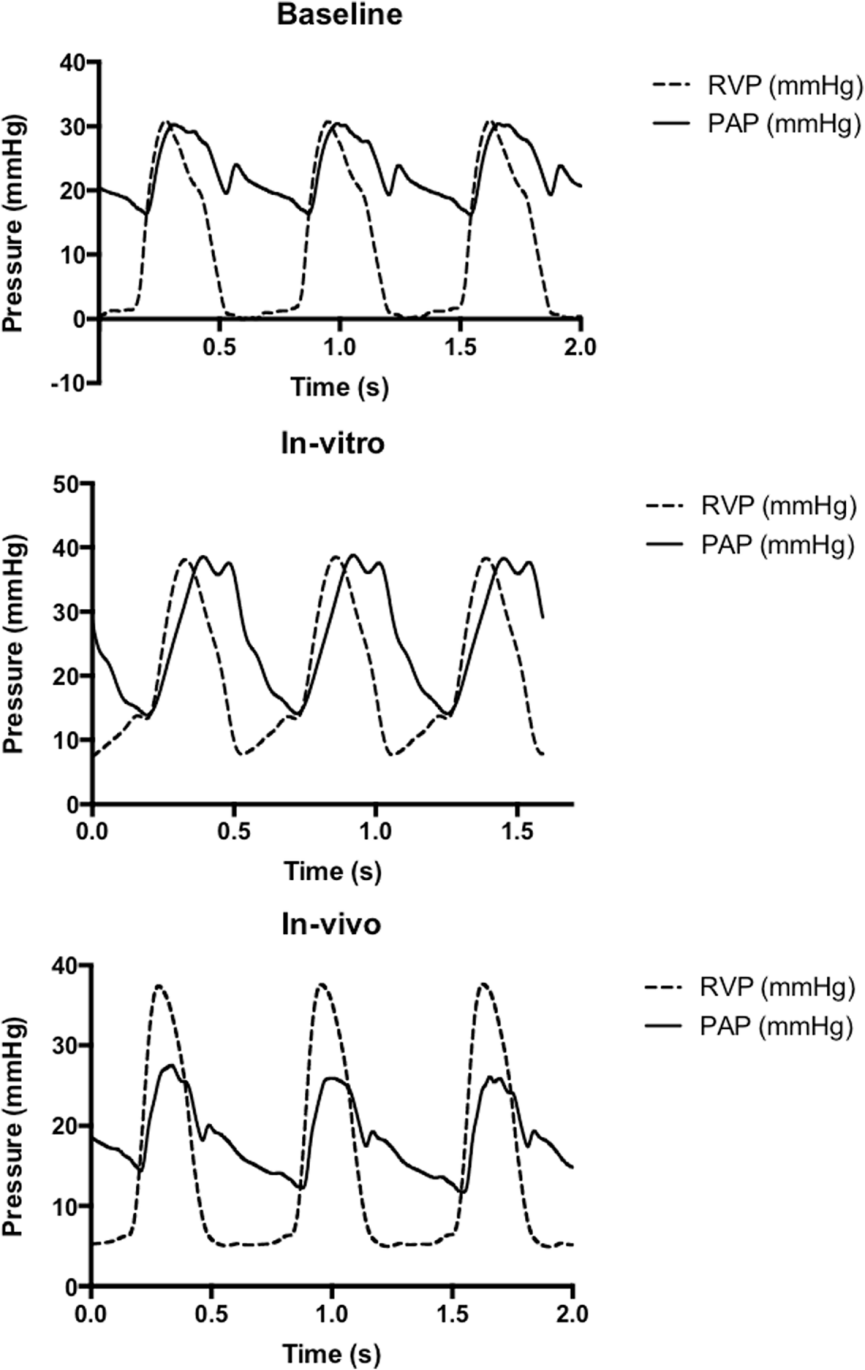
Pressure curves for all three groups. RVP: Right ventricular pressure. PAP: Pulmonary pressure

A peak right ventricular pressure of 37.7 ± 0.3 mmHg was recorded. Simultaneously, a peak pressure of 26.8 ± 1.2 mmHg was found in the distal (native) part of the pulmonary artery, yielding a peak valve gradient of 10.9 ± 1.3 mmHg. As seen in Fig. 4, a diacrotic notch on the pulmonary pressure curve was seen at the time of end-systole, just as seen in the in-vitro setup.

Pressure results for each of the three sub studies are summarized in Table 1.

**Table 1:** Hemodynamics parameters for all three groups

	HR	BP	RVPp	RVPm	PAPp	PAPm	PVG
	*BPM*	*mm Hg*					
Baseline	92	79/48	30.9 ± 0.1	−0.3 ± 0.3	30.4 ± 0.1	16.3 ± 0.2	0.6 ± 0.1
SIS-ECM in-vitro	117	NA	38.2 ± 0.3	7.7 ± 0.2	38.5 ± 0.3	13.7 ± 0.4	− 0.3 ± 0.3
SIS-ECM in-vivo	89	78/30	37.7 ± 0.3	5.0 ± 0.2	26.8 ± 1.2	12.6 ± 1.1	10.9 ± 1.3

#### Hemodynamic parameters for all three groups

All results are reported as the average value measured over 10 consecutive heart-cycles. Standard deviation for the 10 measure points is shown.

*HR: Heart rate. BPM: Beats per minute. BP: systemic blood pressure (measured in the ascending aorta). RVPp: Peak right ventricular pressure. RVPm: Minimum right ventricular pressure. PAPp: Peak pulmonary artery pressure. PAPm: Minimum pulmonary pressure. PVG: Peak Valve Gradient.*

## Discussion

In this study, we successfully demonstrated that it is possible to produce a three cusp pulmonary valve with proximal support that is made entirely from biodegradable materials. Evaluation of the valve in-vitro and in-vivo showed that the valve was fully functional and competent. Pressure measurements revealed a similar or even superior valve pressure gradient compared to current surgical treatments. Gulack and colleagues found a median peak valve gradient of 20 mmHg in a population of 186 human individuals undergoing pulmonary valve replacement [14]. In the experimental chronic study done by Miller and colleagues a gradient of 10 mmHg was seen using a valve design comparable to the present, but without the supporting ring [12]. In our study, with the supporting ring, a peak valve gradient of 11 mmHg was observed.

Comparing the pressure curves from each substudy (Fig. 4), features of the SIS-ECM valve can be observed. The diacrotic notch seen for both the in-vitro and in-vivo measurements indicate that the valve has natural physiological traits.

The basal ring was added to provide stabilization to the annular level in order to prevent narrowing of the valve during systole. Providing some radial strength to the proximal part of the SIS-ECM valve could be useful in cases with pulmonary stenosis based on infundibular obstruction and small pulmonary outlets. The present results suggest that this addition of a ring does not impair the immediate function of the valve compared to the SIS-ECM-only valve design or to what is used in today’s clinic. In fact, the gradient of 10.9 mmHg is considerably less than the 35 mmHg that both the European Association of Echocardiography and American Society of Echocardiography defines as the upper limit for mild pulmonary stenosis [15].

The acute nature of this study does not address whether the basal ring will facilitate creation of a new annulus while at the same time allow growth. As the ring is not closed but discontinued, it should be able to gradually slide apart as the recipient grow, but also degenerate and leave only native tissue and cells behind. Proper timing and correlation between ring degeneration and host cell ingrowth will be crucial for maintaining the annular support.

The pliability of the SIS-ECM tissue and discontinuity of the Lactosorb® creates an interesting potential for catheter based delivery of this valve concept. Further development though is needed, and not within the scope of this study to explore this possibility.

Good long-term durability is absolutely mandatory for this valve design to be of clinical value. As addressed in the background section, the SIS-ECM has already shown promising results on the right-sided circulation 6 month after implantation, although in the tricuspid position [11].

Different materials could be used for the valve. To keep the design and intraoperative manufacturing as simple as possible we used CoreMatrix® that does not need pre-implantation cell seeding. LactoSorb® is mainly used in bone surgery, and has to our knowledge not been used in cardiac surgery yet. However, it has some of the features needed for this valve concept to work. Other materials could potentially be used. Maybe even autologous tissue such as a strip of cartilage.

The crucial aspect is that the valve is possible to manufacture intra-operatively and thereby allow for optimal sizing and patient fit.

## Conclusion

This study should be considered as a proof-of-concept study. It can be concluded that the design works in an acute setting and is not inferior to other currently available alternatives. However, in order to discover the full potential of valve design and its long-term potential, further experiments should be performed in a chronic setup.
